# Impact of COVID-19 crisis on medical care of patients with metastasized uro-oncologic disease under systemic cancer therapy: a multicenter study in German university hospitals

**DOI:** 10.1007/s00345-021-03868-2

**Published:** 2021-11-30

**Authors:** Julian P. Struck, Maike Schnoor, Andrea Schulze, Marie C. Hupe, Tomasz Ozimek, Immanuel A. Oppolzer, Marco J. Schnabel, Maximilian Burger, Christopher Darr, Viktor Gruenwald, Boris Hadaschik, Maximilian Weinke, Hubert Kuebler, Jonas C. Klockenbusch, Markus T. Grabbert, Christian Gratzke, Mario W. Kramer, Alexander Katalinic, Axel S. Merseburger

**Affiliations:** 1 Department of Urology, University Hospital Schleswig-Holstein, Campus Luebeck, Ratzeburger Allee 160, 23538 Luebeck, Germany; 2 Department of Social Medicine and Epidemiology and Department of Cancer Epidemiology, University of Luebeck, Ratzeburger Allee 160, 23562 Luebeck, Germany; 3 Department of Urology, University Hospital of Regensburg, Landshuter Straße 65, 93053 Regensburg, Germany; 4 Department of Urology, Pediatric Urology and Uro-Oncology, University Hospital of Essen, Hufelandstraße 55, 45147 Essen, Germany; 5 Department of Urology and Pediatric Urology, University Hospital of Wuerzburg, Oberduerrbacher Straße 6, 97080 Wuerzburg, Germany; 6 Department of Urology, University Hospital of Freiburg, Hugstetterstraße 55, 79106 Freiburg im Breisgau, Germany

**Keywords:** Uro-oncology, COVID19, COVID-19, SARS-CoV-2, Pandemic, Medical care

## Abstract

**Purpose:**

To date, over 4.2 million Germans and over 235 million people worldwide have been infected with severe acute respiratory syndrome coronavirus 2 (SARS-CoV-2). Uro-oncology (UO) patients are particularly vulnerable but in urgent need of life-saving systemic treatments. Our multicentric study examined the impact of the COVID-19 crisis on the medical care of UO patients in German university hospitals receiving ongoing systemic anti-cancer treatment and to detect the delay of medical care, defined as deferred medical treatment or deviation of the pre-defined follow-up assessment.

**Methods:**

Data of 162 UO patients with metastatic disease undergoing systemic cancer treatment at five university hospitals in Germany were included in our analyses. The focus of interest was any delay or change in treatment between February 2020 and May 2020 (first wave of the COVID-19 crisis in Germany). Statistical analysis of contingency tables were performed using Pearson’s chi-squared and Fisher’s exact tests, respectively. Effect size was determined using Cramér’s V (V).

**Results:**

Twenty-four of the 162 patients (14.8%) experienced a delay in systemic treatment of more than 2 weeks. Most of these received immuno-oncologic (IO) treatments (13/24, 54.2%, *p* = 0.746). Blood tests were delayed or canceled significantly more often in IO patients but with a small effect size (21.1%, *p* = 0.042, *V* = 0.230). Treatment of patients with renal cell carcinoma (12/73, 16.4%) and urothelial carcinoma (7/32, 21.9%) was affected the most.

**Conclusions:**

Our data show that the COVID-19 pandemic impacted the medical care of UO patients, but deferment remained modest. There was a tendency towards delays in IO and ADT treatments in particular.

## Introduction

To date, over 4.2 million people in Germany, over 71 million in Europe and over 235 million people worldwide have been infected with the betacoronavirus known as severe acute respiratory syndrome coronavirus 2 (SARS-CoV-2). The worldwide death toll of the lung disease COVID-19 caused by the virus is up to 4.8 million [1] and it is still rising. With almost 94,000 confirmed deaths and a case fatality ratio of 2.2% [2], Germany seems to be less affected by the crisis than many other countries around Europe like France (over 7 million cases, over 117,000 deaths) or the United Kingdom (over 8 million cases, over 137,000 deaths) and around the globe [2]. Still, it changed daily life of medical professionals and other groups in Germany and around Europe, including several lockdowns of amusement places, swimming pools, churches, restaurants, schools, universities and other meeting places to avoid a spread of infection, severe hygienic measures being taken, and led to numerous overloads of hospital capacity [3].

As in many other disciplines, urooncologic research and care in Germany [4] as well as urologic training, research and care around the globe [5–7] have been affected by the COVID-19 crisis. Planned operations had to be delayed [8], video consultations were introduced [3], special hygienic preparations were taken [3] and the inclusion of potentially vulnerable patients in clinical studies had to be delayed [5, 6]. Recently, our working group reported severe adverse disease outcomes and a decrease in emergency room visits for patients with pyelonephritis in the COVID-19 era [9]. Special care must be taken of our elder, chronically ill, multimorbid patients and people in need of special care as they seem to be particularly susceptible to COVID-19 infections [10, 11]. This applies also to uro-oncology (UO) patients—not only do they bear a higher risk for more severe COVID-19 infection courses, they also suffer directly from an overload of the health system [12]. These patients, especially with metastatic disease, often do not only need systemic cancer therapies that cannot be delayed or which are connected to repetitive outpatient and inpatient hospital care. Their underlying uro-oncologic diseases (urothelial/renal/prostate/testicular/penile cancer) or systemic, immuno-modulating therapies also lead to immunosuppression of long duration to some extent. This risk population is confronted with progression risks caused by delayed therapy on the one hand and the risk of severe COVID-19 infections and higher mortality rates on the other [10, 11].

## Theory

There is still a knowledge gap concerning the impact of the COVID-19 pandemic on this specific patient group in Germany, especially in German university hospitals which often maintain medical care in times of crisis such as the COVID-19 pandemic while other hospitals might reach their supply limits. So far, German data concerning oncologic care during COVID-19 crisis have been mostly raised via web-based surveys [4, 13] or have been provided by German health insurances [14]. Our evaluation of the effects of the COVID-19 pandemic on the medical care of patients with metastasized uro-oncologic disease under systemic cancer therapy in German university hospitals should help to identify the potential undertreatment of this patient cohort. In particular, we tried to answer the following questions:

How does the COVID-19 pandemic change or influence the care of UO patients in university hospitals under ongoing systemic treatment? Is there a significant difference between urologic cancer entities or substance groups? Do delays in systemic treatment due to the COVID-19 crisis have an impact on the prognosis and survival of these patients?

## Patients and methods

Between the beginning of the first COVID-19 wave that struck Germany at the end of January 2020 and the end of this wave in June 2020, a total of 162 UO patients with metastatic disease and at least 18 years of age who received systemic anti-cancer treatment at the university hospitals of Luebeck, Regensburg, Freiburg im Breisgau, Wuerzburg and Essen were included. Data were retrieved from medical records. The treatments were delivered as routine medical care. The patients did not receive any additional therapies, medication or imaging for the purpose of this study. Anti-cancer treatment with chemotherapy and immuno-oncologic (IO) treatments including checkpoint inhibitors (CPI) or androgen deprivation therapies (ADT) were permitted. It was mandatory for the last radiologic restaging to be dated before the beginning of the COVID-19 pandemic in February 2020 to ensure the baseline oncological status before the pandemic. The key clinical and diagnostic parameters assessed were: age, sex, patient’s performance status by measuring patient’s level of functioning in terms of their ability to care for themselves, daily activity, and physical ability using the Eastern Cooperative Oncology Group (ECOG) classification, comorbidities, immunosuppressing treatments, tumor stage and entity, systemic anti-cancer therapy, previous radiation and surgical therapies, SARS-CoV-2 and influenza status, medical care including delays in therapy or restaging of more than 2 weeks. Institutional Review Board (IRB) approval was obtained from the Ethics Committee of the University of Luebeck (no. 20-156). The patient data were evaluated retrospectively and subsequently anonymized from the data source onwards. No additional written consent was required. The primary aim was to detect the delay of medical care, defined as deferred medical treatment or deviation of the follow-up assessment. Delays were measured as deviations from the standard times for uro-oncological therapies, follow-up imaging and follow-up examinations, as listed in the approval studies for the respective substance and in the German and European urological guidelines of the German (DGU) and European (EAU) societies for urology.

As a secondary aim, we tested whether tumor entity or treatment modality was associated. Statistical analysis of contingency tables was performed using Pearson’s chi-squared and Fisher’s exact tests, respectively. Effect size was determined by calculating Cramér’s *V* (V) [15] to evaluate the association between tumor entity or treatment modality and delay in follow-up and treatment, respectively. Effect size was defined as small for *V* = 0.1, medium for *V* = 0.3, and large for *V* = 0.5 [16]. The significance level was set to *α* = 0.05. SPSS v22.0 was used (IBM Corp., Armonk, NY, USA) for all analyses and data management.

## Results

All reported changes regarding aftercare and therapy fell during the period of the first Covid wave and the first Covid-related lockdown in Germany from February 2020 to June 2020. The majority of patients were male (87.6%); median age at the time of inclusion was 68.5 years (range 33–89 years); 81.9% of cases exhibited favorable clinical conditions (ECOG performance status 0–1). More detailed patient characteristics are presented in Table 1. Only 1/49 SARS-CoV-2 PCR tests conducted were reported positive. The patient did receive in-house care because of the SARS-COV-2 infection but without intensive care and he recovered fully.

**Table 1:** Patient characteristics

Characteristics	No change in therapy or follow-up	Any change in therapy or follow-up	Total	*p*-value*
Sex (1 missing value)				
Male	93 (66.0%)	48 (34.0%)	141	0.025
Female	8 (40.0%)	12 (60.0%)	20	
Age (median, range)	67 (33–88)	67.5 (48–89)	67.1 (33–89)	0.202
Diagnosis				
Castration-resistant prostate cancer	22 (66.7%)	11 (33.3%)	33	0.304
Hormone-sensitive prostate cancer	11 (64.7%)	6 (35.3%)	17	
Renal cell carcinoma	41 (56.2%)	32 (43.8%)	73	
UTUC	7 (77.8%)	2 (22.2%)	9	
MIBC	14 (63.6%)	8 (36.4%)	22	
NMIBC	0 (0%)	1 (100%)	1	
Penile carcinoma	2 (100%)	0 (0%)	2	
Testicular cancer	5 (100%%)	0	5	
ECOG Performance status (1 missing value)				
Asymptomatic (0)	36 (58.1%)	26 (41.9%)	62	0.123
Symptomatic but completely ambulatory (1)	46 (65.7%)	24 (34.3%)	70	
Symptomatic, < 50% in bed during the day (2)	9 (50.0%)	9 (50.0%)	18	
Symptomatic, > 50% in bed, but not bedbound (3)	8 (100%)	0 (0%)	8	
Bedbound (4)	2 (100%)	0 (0%)	2	
Death (5)	1 (100%)	0 (0%)	1	

ECOG, Eastern Co-operative Oncology Group, UTUC, upper tract urothelial carcinoma, MIBC, muscle-invasive bladder cancer, NMIBC, non-muscle-invasive bladder cancer

*Chisquare test in categorical variables, t-test in metric variables

**Fig. 1:**
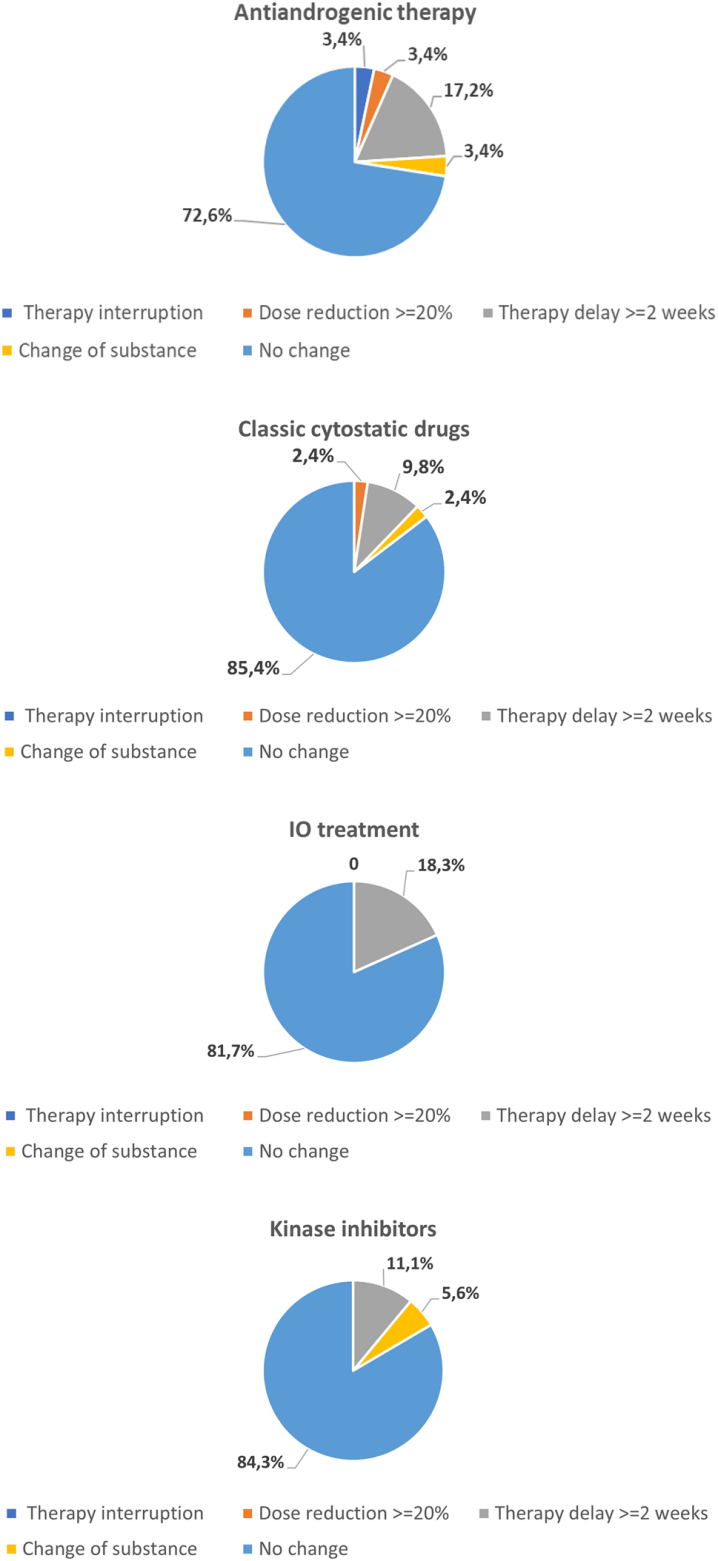
Therapy changes by substance

**Fig. 2:**
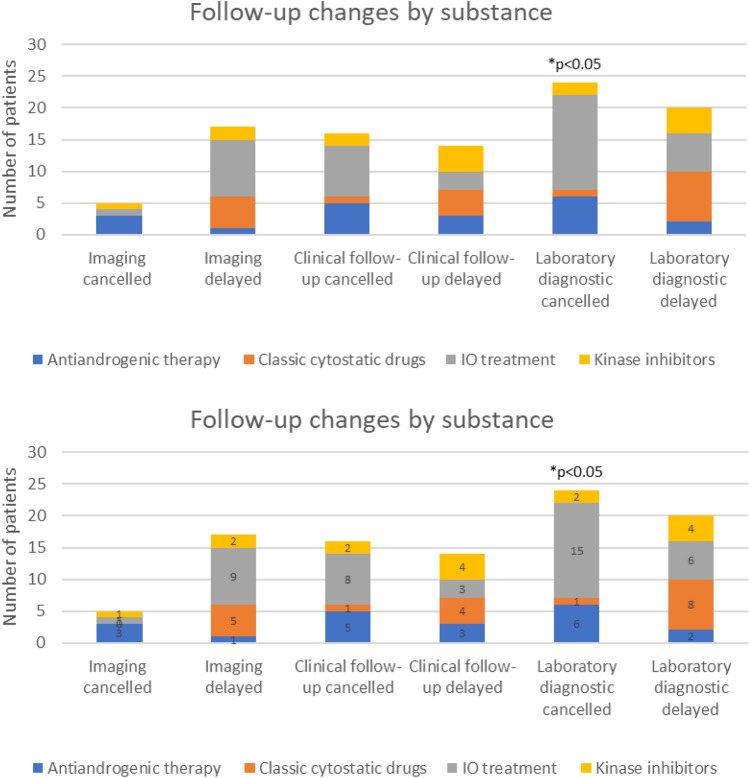
Follow-up changes by substance

**Fig. 3:**
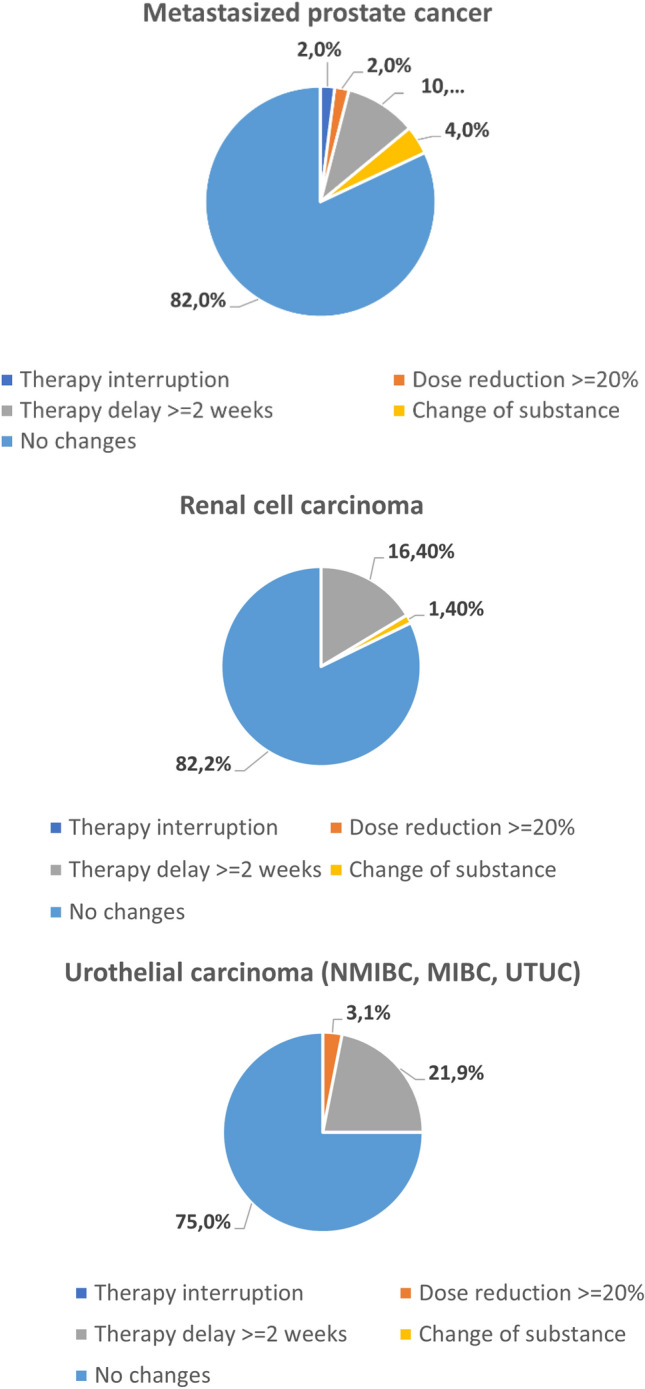
Therapy changes by entity

Our data revealed that 30/161 patients (18.6%) experienced a therapeutic change due to the COVID-19 pandemic (Tables 2 and 3). Of those, 24 (80%) experienced a delay in the systemic treatment of more than 2 weeks; three patients (10%) had a switch in their therapeutic agents. Most of the patients who experienced a delay in systematic treatment received IO treatments (13/24, 54.2%) or ADT (5/24, 20.8%, *p* = 0.746, Fig. 1). In only one case was treatment completely interrupted. 53 (32.3%) patients experienced a change in their follow-up and imaging schedule (Tables 4 and 5). In 17 (32.1%) of these 53 cases, radiologic follow-up had been delayed; in five cases (9.4%) it was cancelled. In 16/53 cases (30.2%), clinical follow-up had been cancelled and in 14/53 cases (26.4%) it was delayed. In 24/53 cases (45.3%), blood tests had been cancelled, significantly more often in IO patients but with a small effect size (21.1%, *p* = 0.042, *V* = 0.230, Fig. 2). 20 of the 53 patients (37.7%) experienced a delay in blood tests. The pandemic influenced the treatment of patients with renal cell carcinoma (RCC) (12/73, 16.4%) and urothelial carcinoma (UC) (7/32, 21.9%) more frequently than in those with prostate cancer (5/50, 10%) or testicular cancer (0%, Fig. 3). Eleven of the 162 patients (6.8%) died due to the progression of cancer.

**Table 2:** Therapy changes among substances

Substance group	*N*	Therapy interruption	Dose reduction ≥ 20%	Therapy delay ≥ 2 weeks	Change of substance
Androgen deprivation therapy	29	1 (3.4%)	1 (3.4%)	5 (17.2%)	1 (3.4%)
Chemotherapy	41	0	1 (2.45%)	4 (9.8%)	1 (2.4%)
IO treatment	71	0	0	13 (18.3%)	0 (0%)
Kinase inhibitors	18	0	0	2 (11.1%)	1 (5.6%)
Parp inhibitors	2	0	0	0 (0%)	0 (0%)
Total	161	1 (0.6%)	2 (1.2%)	24 (14.9%)	3 (1.9%)
*p*-value (Fisher’s exact test)		0.304	0.322	0.746	0.179

IO, immuno-oncologic; Parp, poly(ADP-ribose)-polymerase

**Table 3:** Therapy changes among cancer entities

Cancer entity	*N*	Therapy interruption	Dose reduction ≥ 20%	Therapy delay ≥ 2 weeks	Change of substance
Metastasized prostate cancer	50	1 (2%)	1 (2%)	5 (10%)	2 (4%)
Testicular cancer	5	0	0	0	0
Urothelial carcinoma	32	0	1 (3.1%)	7 (21.9%)	0
Renal cell carcinoma	73	0	0	12 (16.4%)	1 (1.4%)
Penile cancer	2	0	0	0	0
Total	162	1 (0.6%)	2 (1.2%)	24 (14.8%)	3 (1.9%)
*p*-value (Fisher’s exact test, 2-sided)		0.549	0.339	0.587	0.274

**Table 4:** Follow-up changes among substance groups

Substance group	*N*	Imaging cancelled	Imaging delayed	Clinical follow-up cancelled	Clinical follow-up delayed	Laboratory diagnostic cancelled	Laboratory diagnostic delayed
Androgen deprivation therapy	29	3 (10.3%)	1 (3.4%)	5 (17.2%)	3 (10.3%)	6 (20.7%)	2 (6.9%)
Conventional cytostatic drugs	41	0 (0%)	5 (12.2%)	1 (2.4%)	4 (9.8%)	1 (2.4%)	8 (19.5%)
IO treatment	71	1 (1.4%)	9 (12.7%)	8 (11.3%)	3 (4.2%)	15 (21.1%)	6 (8.5%)
Kinase inhibitors	18	1 (5.6%)	2 (11.1%)	2 (11.1%)	4 (22.2%)	2 (11.1%)	4 (22.2%)
Parp inhibitors	2	0 (0%)	0 (0%)	0 (0%)	0 (0%)	0 (0%)	0 (0%)
Total	161	5 (3.1%)	17 (10.6%)	16 (9.9%)	14 (8.7%)	24 (14.9%)	20 (12.4%)
*p*-value (Fisher’s exact test)		0.102	0.672	0.256	0.157	0.042	0.218
Cramér’s *V*		0.215	0.118	0.171	0.198	0.230	0.187

IO, immuno-oncologic; Parp, poly(ADP-ribose)-polymerase

**Table 5:** Follow-up changes among cancer entities

Cancer entity	*N*	Imaging cancelled	Imaging delayed	Clinical follow-up cancelled	Clinical follow-up delayed	Laboratory diagnostic cancelled	Laboratory diagnostic delayed
Metastasized prostate cancer	50	3 (6%)	2 (4%)	5 (10%)	5 (10%)	8 (16%)	3 (6%)
Testicular cancer	5	0	0	0	0	0	0
Urothelial carcinoma	32	0	5 (15.6%)	4 (12.5%)	1 (3.1%)	6 (18.8%)	2 (6.3%)
Renal cell carcinoma	73	2 (2.7%)	10 (13.7%)	7 (9.6%)	9 (12.3%)	11 (15.1%)	15 (20.5%)
Penile cancer	2	0	0	0	0	0	0
Total	162	5 (3.1%)	17 (10.5%)	16 (9.9%)	15 (9.3%)	25 (15.4%)	20 (12.3%)
*p*-value (Fisher exact test, 2-sided)		0.544	0.322	0.946	0.602	0.930	0.107

## Discussion

Chinese observations on SARS-CoV-2-infected cancer patients in three hospitals in Wuhan revealed that 53.6% of patients (15/28) had severe events, with a mortality rate of 28.6% [10]. If the last antitumor treatment was within 14 days, it significantly increased the risk of developing severe events. Kuderer et al. [11] analyzed data from the USA, Canada and Spain from the COVID-19 and Cancer Consortium (CCC19) database between March 17 and April 16, 2020 and found a higher 30-day mortality in prostate cancer patients under systemic treatment.

This possible threat combined with other restraints in patient care due to the COVID-19 crisis led to delays and changes in therapy, as the literature shows. Froehling et al. [4] reported changes in radiologic diagnostics, systemic oncologic treatments and oncologic operations. Satish et al., showed a treatment delay or change in 43% of their breast cancer patients. Other groups revealed dose reductions or discontinuation of systemic cancer treatments [17]. Our data illustrate that 24 (14.8%) patients in the total cohort experienced a delay in the systemic treatment of more than 2 weeks, of whom three patients (10%) had a switch in their therapeutic agents. Most of these patients received IO treatments (13/24, 54.2%) or ADT (5/24, 20.8%, *p* = 0.746). Recent data show an ongoing treatment effect of IO agents [18] after discontinuation, which could explain the highest rate of treatment delays in this group. Existing data indicate that survival rates for intermittent androgen deprivation (IAD) are similar to those for continuous ADT [19], which supports the possibility of treatment changes in this group during the COVID-19 crisis.

In line with several surveys and reports from Germany and other countries [4, 7, 20], our data support a tendency towards delays in the follow-up care of UO patients, mainly during the first 6 weeks of COVID-19-related lockdown from March to mid-May 2020. Our study revealed that IO patients, in particular, experienced delays in follow-up care. Due to the fact that IO treatment agents are often less toxic than classical chemotherapeutic agents and that radiologic response varies among patients [21, 22], follow-up periods are defined less strictly. In contrast to other reports which could not show a relevant delay in laboratory diagnostics, our data illustrate that particularly blood tests had been delayed or cancelled in 43 cases (26.6%), significantly more often in IO patients but with a small effect size (21.1%, *p* = 0.042, *V* = 0.230).

The pandemic influenced the treatment of RCC (12/73, 16.4%) and UC (7/32, 21.9%) patients more frequently than those with prostate cancer (5/50, 10%) or testicular cancer (0%), which might be due to the more invasive treatment in these groups. In general, patients with testicular cancer are often younger than other UO patients and therefore receive more aggressive treatments. This could explain why testicular patients did not experience any delays in therapy or follow-up. Still, it is alarming that two cancer types with a high risk for progression caused by delays [12] were affected the most.

Our data revealed that 11/162 patients (6.8%) died due to the progression of cancer. A recently published population-based study expects an increase in cancer mortality of 5–17% in England over the next 5 years due to a decrease in the presentation of suspected cancer cases and restricted diagnosis during COVID-19-caused lockdown [23]. To measure how and to what extent changes to therapy and follow-up have an impact on treatment and survival outcome in UO patients, more long-term data analyses, e.g. from cancer registers, are needed over the years.

### Countermeasures and solutions

In the following, we present some pragmatic measures that have been implemented in Germany and around the globe to cope with the health emergency related to COVID-19 and to counteract possible under-treatment of cancer patients, although the relative effectiveness of each intervention needs to be further analyzed in large observational studies. An Italian group around Panebianco suggested adapting guideline recommendations and integrating mpMRI in diagnostics for the management of bladder cancer as a risk-adapted strategy during the COVID-19 pandemic [24].

To avoid severe consequences of delayed in-house presentation as we have already reported for emergency units [9], it is necessary to keep in touch with patients during COVID-19-caused lockdowns, especially UO patients who need systemic cancer therapies that cannot be delayed or which are associated with repetitive outpatient and inpatient hospital care. Prior to the COVID-19 crisis, our working group described the possibility of implementing telemedicine in urological care as well as the chances of digitalization for urology [25, 26]. Video consultations could help to avoid delays in UO follow-up and help the urologist to differentiate more easily the cases in which there is an urgent need for in-house consultations as well as those in which follow-up could be possibly delayed. Others have installed an online oncology platform for communicating with cancer patients [3]. Digitalization, especially the use of social media sources for disseminating medical information to a large audience making them valuable campaigning instruments [27] or by providing applications (apps) to optimize follow-up care [28], has the potential to optimize oncologic patient care and in this way it has enormous potential for the field of UO. Therefore, the German health care system needs structured campaigns to speed up digitalization in the near future.

Based on survey results, the German National Association of Urology (DGU e.V.) and the German Association of Uro-Oncologists (d-uo e.V.) [29] as well as the European association (EAU) [30] and other groups [31] have released suggestions of how to maintain oncologic and UO care in COVID-19-caused pandemics. For surgical urologic procedures, prioritization lists have been published and established [29]. Suggestions have been made to favor UO research, to limit recruitment of patients to studies with presumably “practice-changing” results and to replace on-site monitoring with remote monitoring [3, 32].

### Limitations

However, our study is not devoid of limitations. We tried to avoid a possible selection bias by including university centers all over Germany, especially from Southern (Baden-Wuerttemberg and Bavaria) and Western Germany (North Rhine-Westphalia) which, taking into account the total number of infections and deaths, were much stronger affected by the crisis than the rest of the country [33]. Still, none of our patients died from COVID-19 and only one patient got infected by the virus, and he recovered fully afterwards. Our data suggest that the shielding of uro-oncological patients at German university hospitals could have been effective during the first wave of COVID-19. Furthermore, the suggestions released by the national (March 2020) and European (May 2020) associations described earlier could have had a positive impact.

We initially started our analysis in October 2019 to examine not only the first wave of COVID-19 but also the influence of seasonal flu on uro-oncological patients under ongoing system therapy. Since none of our patients contracted the flu and the flu wave in 2019 was relatively mild, we could not determine any effects/deviations and discarded this analysis. All reported changes regarding aftercare and therapy fell during the period of the first COVID-19 wave and the first COVID-related lockdown in Germany from February 2020 to June 2020 and are to be related to this.

Our study does not claim to stand for UO care in the whole country but it demonstrates the impact of concentrating the medical resources of German university hospitals to fight the COVID-19 crisis on the medical care of UO patients. Apart from prioritization, capacity shortages of medical employees, who had to be in quarantine which resulted in shrinking capacity for UO patients on wards and in outpatient departments, had an additional impact on the medical care of UO patients during the first months of the crisis.

## Conclusions

The COVID-19 crisis had an impact on follow-up care in one-third of cases and on systemic treatment in one-fifth. There was a tendency towards delays in IO and ADT treatments in particular. Data prove that the crisis has had an impact on the medical care of UO patients but so far yield no statistically measurable prognostic or survival impact. Long-term follow-up data are needed and will be delivered for further evaluations.

## Data Availability

Access to associated data will be granted on request to the main or senior author.
